# Quantifying Liver Cirrhosis by Extracting Significant Features from MRI T2 Image

**DOI:** 10.1100/2012/343847

**Published:** 2012-06-18

**Authors:** Ming-Hong Hshiao, Po-Chou Chen, Jo-Chi Jao, Yung-Hui Huang, Chen-Chang Lee, Shih-Yu Chao, Li-Wei Lin, Tai-Been Chen

**Affiliations:** ^1^Department of Radiology, Chang Gung Memorial Hospital—Kaohsiung Medical Center, Kaohsiung City 83301, Taiwan; ^2^Department of Medical Imaging and Radiological Sciences, I-Shou University, Kaohsiung City 84001, Taiwan; ^3^Department of Medical Imaging Technology, Shu Zen College of Medicine and Management, Kaohsiung City 82144, Taiwan; ^4^Department of Biomedical Engineering, I-Shou University, Kaohsiung City 84001, Taiwan; ^5^Department of Medical Imaging and Radiological Sciences, Kaohsiung Medical University, Kaohsiung City 80708, Taiwan; ^6^The School of Chinese Medicine for Post-Baccalaureate, I-Shou University, Kaohsiung City 84001, Taiwan

## Abstract

Most patients with liver cirrhosis must undergo a series of clinical examinations, including ultrasound imaging, liver biopsy, and blood tests. However, the quantification of liver cirrhosis by extracting significant features from a T2-weighted magnetic resonance image (MRI) provides useful diagnostic information in clinical tests. Sixty-two subjects were randomly selected to participate in this retrospective analysis with assigned to experimental and control groups. The T2-weighted MRI was obtained and to them dynamic adjusted gray levels. The extracted features of the image were standard deviation (SD), mean, and entropy of pixel intensity in the region of interest (ROI). The receiver operator characteristic (ROC) curve, 95% confidence intervals, and kappa statistics were used to test the significance and agreement. The analysis of area under ROC shows that SD, mean, and entropy in the ROI were significant between the experimental group and the control group. Smaller values of SD, mean, and entropy were associated with a higher probability of liver cirrhosis. The agreements between the extracted features and diagnostic results were shown significantly (*P* < 0.001). In this investigation, quantitative features of SD, mean, and entropy in the ROI were successfully computed by the dynamic gray level scaling of T2-weighted MRI with high accuracy.

## 1. Introduction

The prevalence of types B and C hepatitis globally in 2008 was 8%, according to reports of the WHA (World Hepatitis Alliance). Eighty percent of patients infective type C hepatitis will become carriers of liver toxicity [1, 2]. Of the carriers of infective type C hepatitis, 33.3% suffered from liver cirrhosis in two decades and 7% of instances of liver cirrhosis resulted in liver cancer [2, 3]. The hepatitis virus was examined and assigned score by Child-Pugh system, as presented in Table 1 [4, 5]. The high score indicates a high probability of liver cirrhosis. The regular examination of liver function involves testing by blood biomarkers, biopsying of liver tissue, imaging by ultrasound, and imaging by magnetic resonance [6–10]. One successful test for the type C hepatitis virus was used for the gene marker of IL28B [11, 12]. However, the variety of textures and structures of a liver cannot be described and visualized by the gene marker. Liver biopsy is the gold standard for diagnosis and classification of liver cirrhosis. In practice, limited sampling locations, bleeding, and the possibility of infection are the main disadvantages of a liver biopsy. Biopsy has been determined to have morbidity and mortality rates of 3% and 0.03%, respectively, [13]. Identifying cirrhotic tissue is difficult because only 1/50000 of the liver tissue is sampling in a biopsy. Therefore, the rate of negative diagnosis of liver cirrhosis is artificially increased [14]. The advantages of sonographic imaging are that it is noninvasive, does not involve radiation, is fast, and is safe for patients. Ultrasonic imaging has become a popular modality for diagnosing liver functions because it yields images in real time. However, the fundamental problem of ultrasound images is their poor quality, mostly caused by multiplicative speckle noise, and their dependence on the operator [15]. Speckle is a particular kind of noise that affects images that are obtained using coherent imaging systems, such as ultrasonic systems. Errors in date transmission cause “salt and pepper noise” [16]. The poor image quality that is caused by the scattering effects associated with ultrasound speckle limit the rates of positive diagnoses of liver cancer and cirrhosis. The high spatial resolution of MRI provides a much better image of the structure of soft tissues, and the process is irradiative. MRI of the liver is currently the most popular diagnostic tool. However, lack of experience of image-based diagnostics can make a diagnostician less able to classify liver function. The signal-to-noise ratio (SNR) and contrast-to-noise ratio (CNR) are utilized in the quantitative analysis of hepatocellular carcinoma and cirrhotic liver MRI. The size and location of the ROI affect the accuracy of the determination of SNR and CNR [17, 18]. In this investigation, to improve the accuracy, feasibility, reliability, and stability of the proposed method, the selected ROI was the whole of the liver, including both left and right lobes. Meanwhile, the quantitative value (QV) of extracted features were estimated as the using of a confidence interval (CI) of 95%. *P* < 0.05 indicates statistical significance. 

## 2. Method and Materials

### 2.1. Patients

The retrospective study was performed from March 2010 to July 2011. A total of 62 patients were involved and were assigned to the experimental (abnormal) and control (normal) groups. The thirty-one patients who had liver cirrhosis (nine female and 22 male) belonged to experimental group. They were aged 41–80 years and had clinical Child-Pugh scores of greater than 10. The control group comprised 31 patients (14 female and 17 male), aged 19–56 years. They all had glutamic oxaloacetic transaminase (GOT) levels of less than 35 *μ*/L and glutamic pyruvic transaminase (GPT) levels of less than 40 *μ*/L and none had a medical history of virus hepatitis or negative liver function.

### 2.2. Acquisition of Imaging Data

All of the patients were imaged by magnetic resonance scanning with a body coil (Philips 1.5 T MRI SENSE). The intensity of the magnetic field was set to 1.5 tesla (T); the pulse sequence was TSE; TR was 623 ms; TE was set to 80 ms; the flip angle was 900; the TSE factor was 68; the NSA was unity.The acquisition time was approximately 12–22 s. The T2-weighted MR images (scanned using TSE) of all patients were stored in DICOM format (version 3.0) and for subsequent quantitative analysis and extraction of features of the image of the liver. The Section 2.3. describes the acquisition of the images and the extraction of features.

### 2.3. Image Processing and Extracting Features from Images of Liver

**Figure 1:**
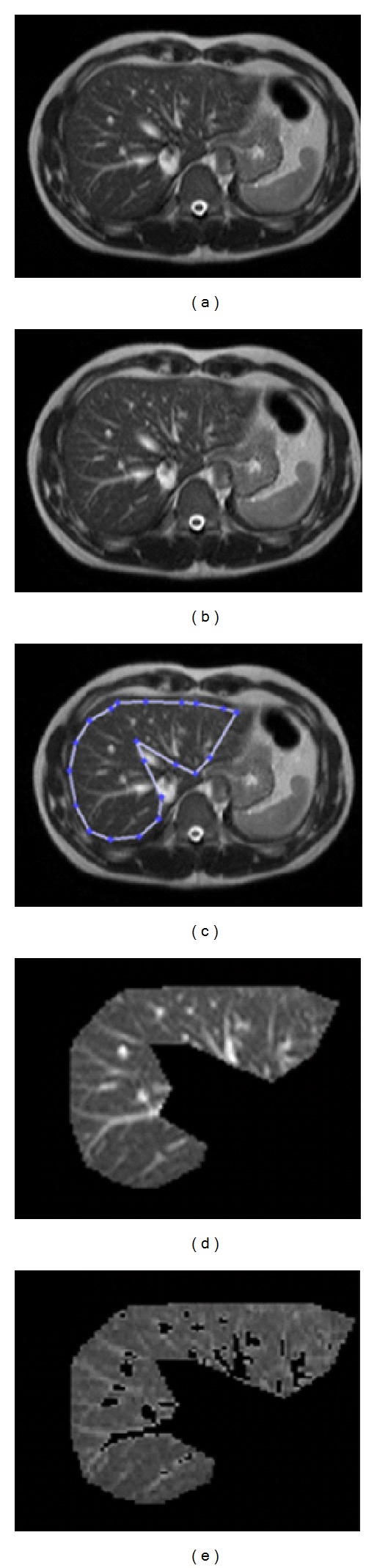
(a) MRI T2-weighted image (original scale), (b) MRI T2-weighted image on altered scale, (c) selected ROI of liver, (d) magnified ROI of liver, and (e) magnified ROI of liver after filtering out of artery and portal veins using Otsu's algorithm.

To increase the heterogeneity and contrast of T2 images, the dynamic range of the c gray level in MRI T2-weighted images was scaled from (0, 4095) to (0, 63) as the first step. This process is also called normalization or dynamic gray level scaling. It is very effective for increasing the variety of pixel intensities in MRI T2-weighted images. The second step was to select a part of the liver in the T2-weighted image. The selection of the ROI of the liver referred to the left and right lobes of the liver in a single slice of the T2 image. The third step was the automatic segmentation (Otsu's method) to filter out the arteries and portal veins from the ROI of the liver. The final step was to extract the features of the ROI, including the mean, standard deviation (SD), and entropy of pixel intensity.  Figure 1 presents the steps of the analysis.

### 2.4. Statistical and Quantitative Analyses

The *t*-test was conducted to study the significance or otherwise of the difference between the SDs, means, and entropies of the pixel intensities of the normal (control) and abnormal (experimental) groups. The receiver operating characteristic curve (ROC) was used to evaluate the sensitivity and specificity of the extracted features for normal and abnormal groups. Also, the quantitative values (QV) of the extracted features were determined at the convex point of the AUC. The kappa statistics, the strength of the agreement between the diagnostic examination, and the extracted features were determined. The PPV (positive predictive value), NPV (negative predictive value), accuracy, sensitivity, and specificity were utilized to represent the performance of quantification in terms of the SD, mean, and entropy of pixel intensity in the ROI.

## 3. Results

**Figure 2:**
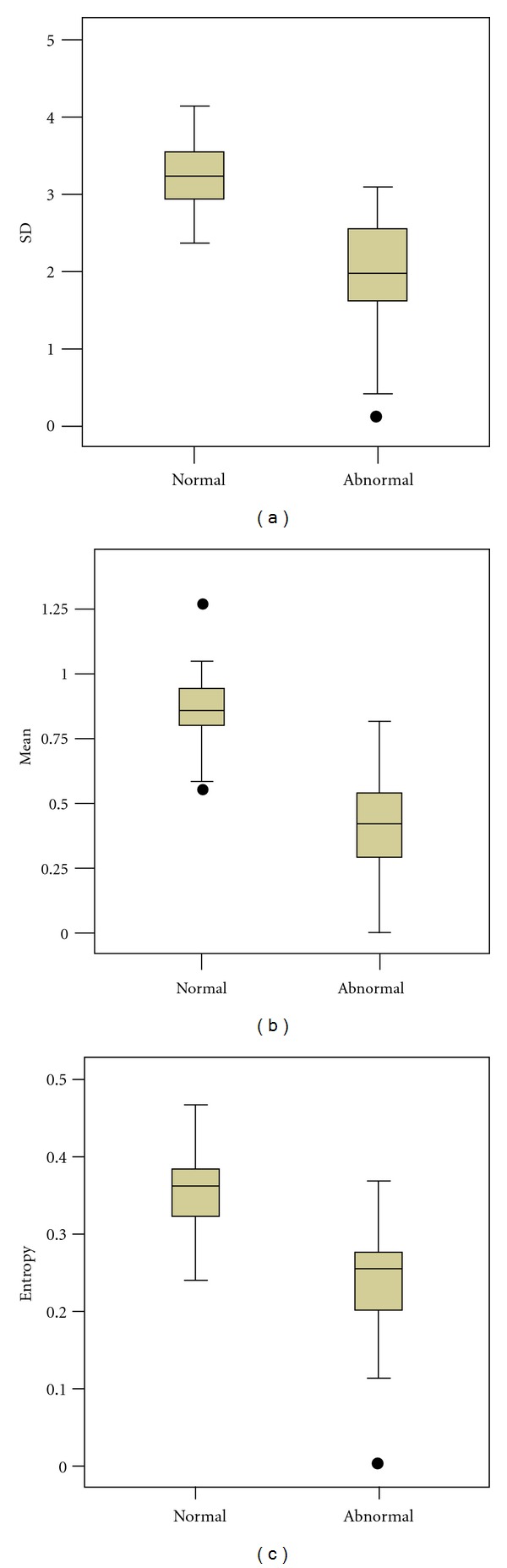
Boxplots of normal and abnormal groups SD, mean, entropy, and body weight compared with reference line.

**Figure 3:**
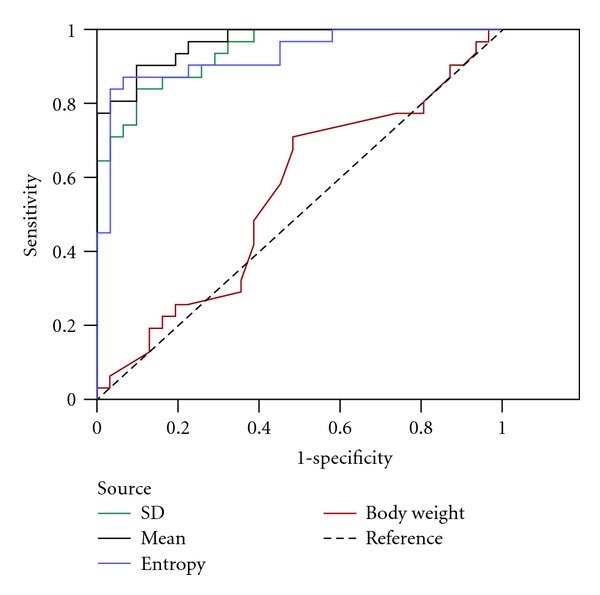
The ROC of extracted features of image (SD, mean, entropy of pixel intensity in the ROI) and body weight associated with normal and abnormal groups.

Figure 2 presents boxplots of the SD, mean, and entropy of pixel intensity for the normal and abnormal groups. The differences between normal and abnormal groups are shown.  Figure 3 shows the ROC of SD, mean, and entropy of pixel intensity as well as the patient weight associated with the classified normal and abnormal groups. The SD, mean, and entropy of pixel intensity in the ROI were enabled, the performance of normal and abnormal groups are to be determined. Age and body weight had no direct effect on either of the two groups.  Table 2 presents the descriptive statistics of age, body weight, and extracted features (SD, mean, and entropy) between groups. 95% CI of all extracted features present lack of overlapping intervals between normal and abnormal groups. Therefore, the SD, mean, and entropy of pixel intensity in selected ROI can be used to perform classification of normal and abnormal groups. Meanwhile, the *P* values in a *t*-test of extracted features reveal statistically significant differences between the normal and abnormal groups (*P* < 0.001).  Table 3 presents the QV determined by the convex point of ROC for SD, mean, and entropy, which are 2.8, 0.7, and 0.3, respectively. The sensitivity, specificity, accuracy, PPV, and NPV all exceeded 0.8 for classification of normal and abnormal groups by applied SD, mean, and entropy. The strengths of the agreements of SD, mean, and entropy with diagnostic results, determined from the kappa statistics were 0.677, 0.774, and 0.806, respectively. The strength of the agreements, specified by the kappa coefficient was high in this investigation. AUC is a widely used measure of accuracy of classification. Higher values of AUC indicate better classification. In this investigation, the AUC values of SD, mean, and entropy all exceeded 93% (*P* < 0.001). The *t*-test, 95% CI, and AUC revealed that SD, mean, and entropy of pixel intensity in ROI can be used to distinguish between normal and abnormal groups with high accuracy and statistical significance.

## 4. Conclusion

MRI is safe, fast, and noninvasive diagnostic tool with high spatial resolution. In this study, a new concept of dynamic gray level scaling and the filtering out of the artery and portal veins in the ROI was successfully applied to MRI of liver cirrhosis. The extracted features of the MRI T2-weighted image provide a quantitative measure of the extent of liver cirrhosis. The strength of the agreement between extracted features and diagnostic results determined using the kappa statistics, the determination of performance determined using AUC, and the proposed method can be used accurately to identify a cirrhotic or normal liver. Although the clinical diagnosis of liver cirrhosis has, to date, required a series of tedious and dangerous medical tests, the proposed method and extracted features may provide useful quantitative information concerning cases of liver cirrhosis. The proposed method integrated image processing, automatic segmentation, and image mining with the use of medical reports. The 95% CI and AUC were utilized to identify significant features. The identification of liver cirrhosis was carried out by extracting significant features from an MRI T2-weighted image. The most significant features, the SD, mean, and entropy of pixel intensity in the ROI, were suggested and investigated. Small values of SD, mean, and entropy indicate homogenous pixel intensity and a high probability of serious liver cirrhosis. SD, mean, and entropy of less than 2.8, 0.7, and 0.3 herein indicated an abnormal liver MRI. The SD and entropy were used to quantify the variation of pixel intensity. Pixel intensity is commonly uniform in MRIs of serious liver cirrhosis. Therefore, the SD and entropy thereof are small. However, the mean intensity represents the image intensity. A small mean typically indicates a stiffness and hardness of liver tissue. Hence, mean is an important factor identifying a normal or an abnormal liver. Age and body weight indirectly affect liver cirrhosis.

## Figures and Tables

**Table 1:** Child-Pugh scores for diagnostic hepatitis virus.

Measure	1 point	2 points	3 points
Total bilirubin (mg/dL)	<2	2-3	>3
Serum albumin (g/L)	>35	28–35	<28
INR	<1.7	1.7–2.2	>2.2
Ascites	None	Moderate	Severe
Hepatic encephalopathy	None	1-2 grade	3-4 grade

**Table 2:** Descriptive statistics of age, body weight, and extracted features (SD, mean, and entropy of pixel intensity in the ROI). *P* values from *t*-test reveal statistically significant differences between extracted features of images of normal and abnormal groups.

Variable	Category	Mean	95% C.I.		SD	Min.	Max.	*P*
			Lower	Upper				
Age	Normal	29.419	26.339	32.500	8.398	19.000	56.000	NA
	Abnormal	56.000	52.066	59.934	10.724	37.000	80.000	
Weight	Normal	67.161	62.203	72.120	13.518	46.000	120.000	NA
	Abnormal	65.452	60.642	70.261	13.112	45.000	114.000	
Entropy	Normal	0.356	0.339	0.373	0.047	0.240	0.467	2.28E−10
	Abnormal	0.233	0.205	0.262	0.077	0.002	0.368	
Mean	Normal	0.865	0.804	0.925	0.164	0.558	1.263	6.82E−14
	Abnormal	0.410	0.335	0.484	0.203	0.001	0.815	
SD	Normal	3.253	3.086	3.421	0.456	2.372	4.138	2.97E−11
	Abnormal	1.969	1.693	2.245	0.753	0.112	3.100	

**Table 3:** The sensitivity, specificity, accuracy, PPV, NPV, kappa statistics, AUC, and *P* value of AUC are listed for QV of 2.8, 0.7, and 0.3 with respective to SD, mean, and entropy of pixel intensity in the ROI.

Performance	SD (2.8)	Mean (0.7)	Entropy (0.3)
Sensitivity	0.839	0.875	0.963
Specificity	0.839	0.900	0.857
Accuracy	0.839	0.887	0.903
PPV	0.839	0.875	0.963
NPV	0.839	0.900	0.857
Kappa	0.677	0.774	0.806
AUC	0.941	0.966	0.930
*P* value of AUC	2.49E−09	2.97E−10	5.83E−09
